# Breast Milk Virome and Bacterial Microbiome Resilience in Kenyan Women Living with HIV

**DOI:** 10.1128/mSystems.01079-20

**Published:** 2021-03-16

**Authors:** Rabia Maqsood, Joshua B. Reus, Lily I. Wu, LaRinda A. Holland, Ruth Nduati, Dorothy Mbori-Ngacha, Elizabeth Maleche-Obimbo, Emily R. Begnel, Soren Gantt, Ednah Ojee, Dalton Wamalwa, Grace John-Stewart, Jennifer Slyker, Dara A. Lehman, Efrem S. Lim

**Affiliations:** a Center for Fundamental and Applied Microbiomics, Biodesign Institute, Arizona State University, Tempe, Arizona, USA; b Department of Paediatrics and Child Health, University of Nairobi, Kenyatta National Hospital, Nairobi, Kenya; c United Nations Children’s Fund (UNICEF), Nairobi, Kenya; d Department of Global Health, University of Washington, Seattle, Washington, USA; e Département de Microbiologie, Infectiologie et Immunologie, Université de Montréal, Centre de Recherche du CHU Ste-Justine, Montréal, Québec, Canada; f Department of Epidemiology, University of Washington, Seattle, Washington, USA; g Division of Human Biology, Fred Hutchinson Cancer Research Center, Seattle, Washington, USA; h School of Life Sciences, Arizona State University, Tempe, Arizona, USA; Northern Arizona University

**Keywords:** breast milk, CMV, HIV, microbiome, virome

## Abstract

Breast milk is nutritionally and immunologically beneficial in early life but is also a potential source of infection. Little is known about breast milk microbiota of women living with HIV (WLHIV), the impact of severe immunosuppression, and the contribution to mortality of HIV-exposed infants. Here, we performed metagenomic sequencing to characterize the bacterial microbiome and DNA virome of breast milk samples at 1 month postpartum from Kenyan WLHIV who were not receiving combination antiretroviral therapy (cART), 23 women with CD4 counts of <250 and 30 women with CD4 of >500; and additionally, 19 WLHIV with infants that lived and 26 WLHIV with infants that died during the first 2 years of life were included. We found that breast milk bacterial microbiomes in this study population were highly diverse but shared a core community composed of the *Streptococcaceae*, *Staphylococcaceae*, *Moraxellaceae*, and *Eubacteriaceae* families. The breast milk virome was dominated by human cytomegalovirus (CMV) and included the bacteriophage families *Myoviridae*, *Siphoviridae*, and *Podoviridae*. Bacterial microbiome and virome profiles and diversity were not significantly altered by HIV immunosuppression, as defined by a CD4 of <250. CMV viral load was not associated with maternal CD4 counts or infant mortality. In conclusion, we show that the core bacterial and viral communities are resilient in breast milk despite immunosuppression in WLHIV.

**IMPORTANCE** Breastfeeding plays an important role in seeding the infant gut microbiome and mammary health. Although most studies focus on the diverse breast milk bacterial communities, little is known about the viral communities harbored in breast milk. We performed the first breast milk virome study of an HIV population. In this study cohort of Kenyan women living with HIV from the pre-antiretroviral therapy era, we found that breast milk harbors a core bacterial microbiome and a virome dominated by human cytomegalovirus. The virome and bacterial microbiome were not substantially altered by immunosuppression or associated with infant mortality. Together, these findings indicate resilience of the microbial community in breast milk compartmentalization. These findings advance out fundamental understanding of the breast milk core microbiome and virome interactions in the context of HIV disease.

## INTRODUCTION

Breastfeeding confers many benefits to an infant, including optimal nutrition, protective immunity through antibodies, and seeding of the gut microbiota (1–3). Breast milk (BM) harbors a diverse community of bacteria (bacterial microbiome) that contributes to the establishment of healthy infant gut flora (1, 4–10). The small number of studies of the human bacterial microbiome in breast milk conducted to date have revealed common “core” bacterial taxa, comprising an array of bacterial families, including *Streptococcaceae* and *Staphylococcaceae*, consistently detected in the breast milk of women across multiple geographic regions (11–14). For example, even though women in rural Ethiopia had abundant *Rhizobium* in their breast milk, they still shared a high abundance of core taxa with women from other countries, such as Spain and Kenya (11). In addition, it has been shown that the bacterial taxa in breast milk and infant stool are highly correlated in early life (9), and thus alterations in the breast milk microbiome are likely to impact the establishment of a healthy infant gut microbiome.

Relatively little is known about the community of viruses (virome) in breast milk. A study of healthy U.S. women found that the majority of the viruses detected in breast milk were bacteriophages from the *Myoviridae*, *Siphoviridae*, and *Podoviridae* families, with very few eukaryotic viruses reported (15). While a relatively small number of pathogenic viruses are known to be transmitted through human milk (including HIV, cytomegalovirus [CMV], and human T lymphotrophic virus type 1 [HTLV-1]) (16–18), these and other viruses may impact both the infant bacterial microbiome and virome through inflammation and immune modulation of the breast milk or infant gut, with subsequent relevance for child outcomes. Studies from women in Italy and the United States found evidence of mother-to-infant virome transmission, as breast milk and stool viromes from mother-infant pairs shared significant homology of bacteriophages (15, 19). Alterations in the breast milk virome may thus influence the establishment of both the infant virome and bacterial microbiome early in life, which could have long-term health consequences (20–24).

Emerging studies indicate that HIV disease has profound effects on microbiota. Immunodeficiency in progressive HIV infections is associated with alterations in the enteric bacterial microbiome and virome (25). For example, immunosuppression among individuals living with HIV (CD4 <200) has been observed to be discriminately associated with bacterial families such as *Enterobacteriaceae* and significantly more *Adenoviridae* in their virome (25). Although one study suggests that women living with HIV (WLHIV) and HIV-uninfected women have similar breast milk bacterial microbiomes (14), the effects of immunosuppression or antiretroviral therapy (ART) on the breast milk bacterial microbiome and virome are not well understood. Given the scarcity of data, it is important to characterize the breast milk bacterial microbiome and virome, particularly in populations with high infant mortality, such as sub-Saharan Africa and populations living with HIV. Based on the low risk of HIV transmission among virally suppressed women, and the nutritional and immunologic benefits of breast milk, current World Health Organization (WHO) guidance recommends women living with HIV (WLHIV) in low-resource settings exclusively breastfeed their infants for 6 months and continue to breastfeed for 1 year or longer as they receive combination antiretroviral therapy (cART) (26). Hence, in the current era of HIV treatment and prevention, HIV-exposed uninfected (HEU) infants will continue to be exposed to a myriad of viruses (other than HIV) in maternal milk. It remains unclear whether maternal HIV infection elicits alterations in the breast milk virome and microbiome or whether changes in the breast milk virome impact infant health outcomes. We used next-generation sequencing to test the hypothesis that severe immunosuppression alters the breast milk DNA virome and bacterial microbiome in WLHIV and that breast milk virome diversity may be associated with mortality in HIV-infected infants.

## RESULTS

### Comparison of bacterial microbiome and virome diversity between women with high and low CD4 counts.

**FIG 1:**
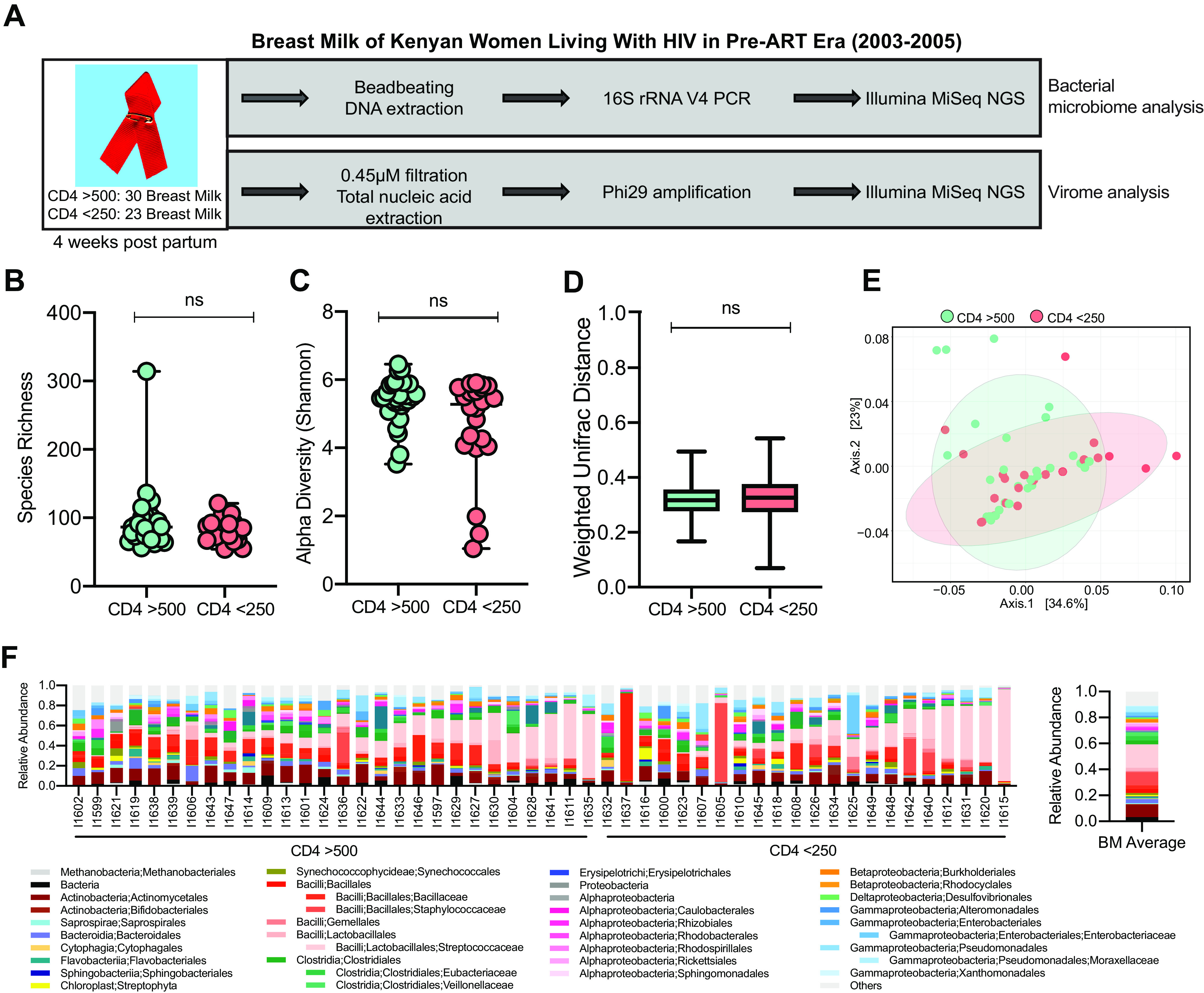
Bacterial microbiome analysis of BM samples from WLHIV by immunosuppression. (A) Overview of study design. (B) Richness of bacterial ASVs in BM samples (median with range). Statistical significance assessed by Mann-Whitney test. (C) Alpha diversity of bacterial ASVs in BM samples (median with range). Statistical significance assessed by Mann-Whitney test. (D) Weighted UniFrac pairwise comparison between samples (median with range). Statistical significance assessed by Mann-Whitney test. (E) PCoA plot of weighted UniFrac distances. Colors represent CD4 count groups. (F) Individual and average relative abundance of bacteria at order and family level in BM samples.

We performed bacterial microbiome 16S rRNA gene sequencing and DNA virome metagenomic sequencing on breast milk samples collected from Kenyan WLHIV at 1 month postpartum as follows: 30 women with CD4 counts of >500 cells/mm^3^ and 23 with CD4 counts of <250 cells/mm^3^ (Fig. 1A, Table 1). As was the standard of care at that time of the original study (2003 to 2005), mothers were given short-course zidovudine during their last trimester to reduce the risk of mother-to-child transmission but received no other cART during follow-up. As expected, the median plasma and breast milk HIV RNA levels were significantly lower in women with CD4 counts of >500 than counts of <250 (plasma, 31,783 versus 455,400 copies/ml [*P* < 0.0001]; BM, 142.5 versus 5,380 copies/ml [*P* < 0.0001]; Fig. S1A and B).

**TABLE 1:** Characteristics of Kenyan WLHIV CD4 cohort

Maternal characteristics^*a*^	Data for samples		
	CD4 > 500 (*n* = 30)	CD4 < 250 (*n* = 23)	*P* value
Maternal age (yr)			
Mean (SD)	23.90 (4.04)	27.48 (5.2)	
Median (IQR)	23 (6.3)	26 (8)	0.012
CD4 counts			
Mean (SD)	832.6 (291.3)	158 (62.2)	
Median (IQR)	782 (368)	170 (102)	<0.0001
CD8 counts			
Mean (SD)	1,263 (498.2)	1,220 (576.8)	
Median (IQR)	1,187 (714.7)	1,066 (758)	0.67
BM HIV viral load (copies/ml)			
Mean (SD)	2,024 (5,856)	30,135 (56,151)	
Median (IQR)	142.5 (266.3)	5,380 (36,930)	<0.0001
Plasma HIV viral load (copies/ml)			
Mean (SD)	157,518 (448,113)	1,136,291 (1,720,360)	
Median (IQR)	31,783 (101,940)	455,400 (1,086,650)	<0.0001

a IQR, interquartile range.

10.1128/mSystems.01079-20.1 FIG S1 Supplemental analysis of BM samples from WLHIV with CD4 of >500 and CD4of <250. (A) Plasma HIV viral load for CD4 of >500 and CD4 of <250 (median with CI). Statistical significance assessed by Mann-Whitney test. (B) Breast milk HIV viral load for CD4of >500 and CD4 of <250 (median with CI). Statistical significance assessed by Mann-Whitney test. (C) Unweighted UniFrac distance box plot for CD4 of >500 and CD4 of <250 groups (median with Tukey). Statistical significance assessed by Mann-Whitney test. (D) PCoA of unweighted UniFrac distances of CD4 of >500 and CD4 of <250 groups. Colors represents CD4 count groups. (E) linear discriminant analysis (LDA) scores of discriminating ASVs between CD4 of >500 and CD4 of <250. (F) Average relative abundance of discriminating ASVs in CD4 of >500 and CD4 of <250 groups. (G) Heat map of discrimination ASVs in CD4 of >500 and CD4 of <250 samples. (H) Unweighted Bray-Curtis PCoA of VirSorter-identified contigs. Colors represents CD4 count groups. (I) Detected qPCR copy numbers of CMV for CD4 of >500 and CD4 of <250 (median with CI). Statistical significance assessed by Mann-Whitney test. (J) Alpha diversity of viral species in BM samples excluding CMV (median with CI). Statistical significance assessed by Mann-Whitney test. (K) Detected qPCR copy numbers of Anellovirus (median with CI). Statistical significance assessed by Mann-Whitney test. (L) LDA scores of discriminating viruses between CD4 of >500 and CD4 of <250. (M) Average relative abundance of discriminating viruses in CD4 of >500 and CD4 of <250 groups. (N) Heat map of discrimination viruses in CD4 of >500 and CD4 of <250 samples. Download FIG S1, PDF file, 0.7 MB. Copyright © 2021 Maqsood et al. 2021 Maqsood et al. https://creativecommons.org/licenses/by/4.0/ This content is distributed under the terms of the Creative Commons Attribution 4.0 International license.

To determine whether immunosuppression due to more severe HIV infection alters the breast milk bacterial microbiome, we compared breast milk samples with sufficient sequencing depth (see Materials and Methods) between women with CD4 counts of >500 (*n* = 28) to those with <250 (*n* = 22). The median bacterial microbiome sequence depth between the two groups was not significantly different (20,450 versus 21,875 average reads/sample for CD4 of >500 versus <250, respectively; *P* = 0.59). Women with high and low CD4 counts had similar bacterial amplicon sequence variant (ASV) richness (median, 86.50 versus 80 for CD4 of >500 and <250, respectively; *P* = 0.65), Shannon alpha diversity (median, 5.47 versus 5.27 for CD4 of >500 and <250; *P* = 0.13), and weighted UniFrac distances (median, 0.32 versus 0.33 for CD4 of >500 and <250; *P* = 0.17; Fig. 1B to D). In addition, the weighted principal-coordinate analysis (PCoA) did not show clustering by group (Fig. 1E). While the unweighted UniFrac distance was significantly higher in breast milk from women with CD4 of >500 than CD4 of <250 (median, 0.67 versus 0.64; *P* < 0.0001; Fig. S1C), the unweighted UniFrac PCoA plot showed four distinct clusters, all of which had samples from both CD4 groups (Fig. S1D). Both groups had high abundance of *Streptococcaceae* (16.6% in CD4 of >500 and 20% in CD4 of <250), *Staphylococcaceae* (6.8% and 14.2%), and *Actinomycetales* (10.6% and 7.7%) (Fig. 1F). Potentially discriminant bacterial taxa by CD4 group were rare and very low in abundance (Fig. S1E to G).

**FIG 2:**
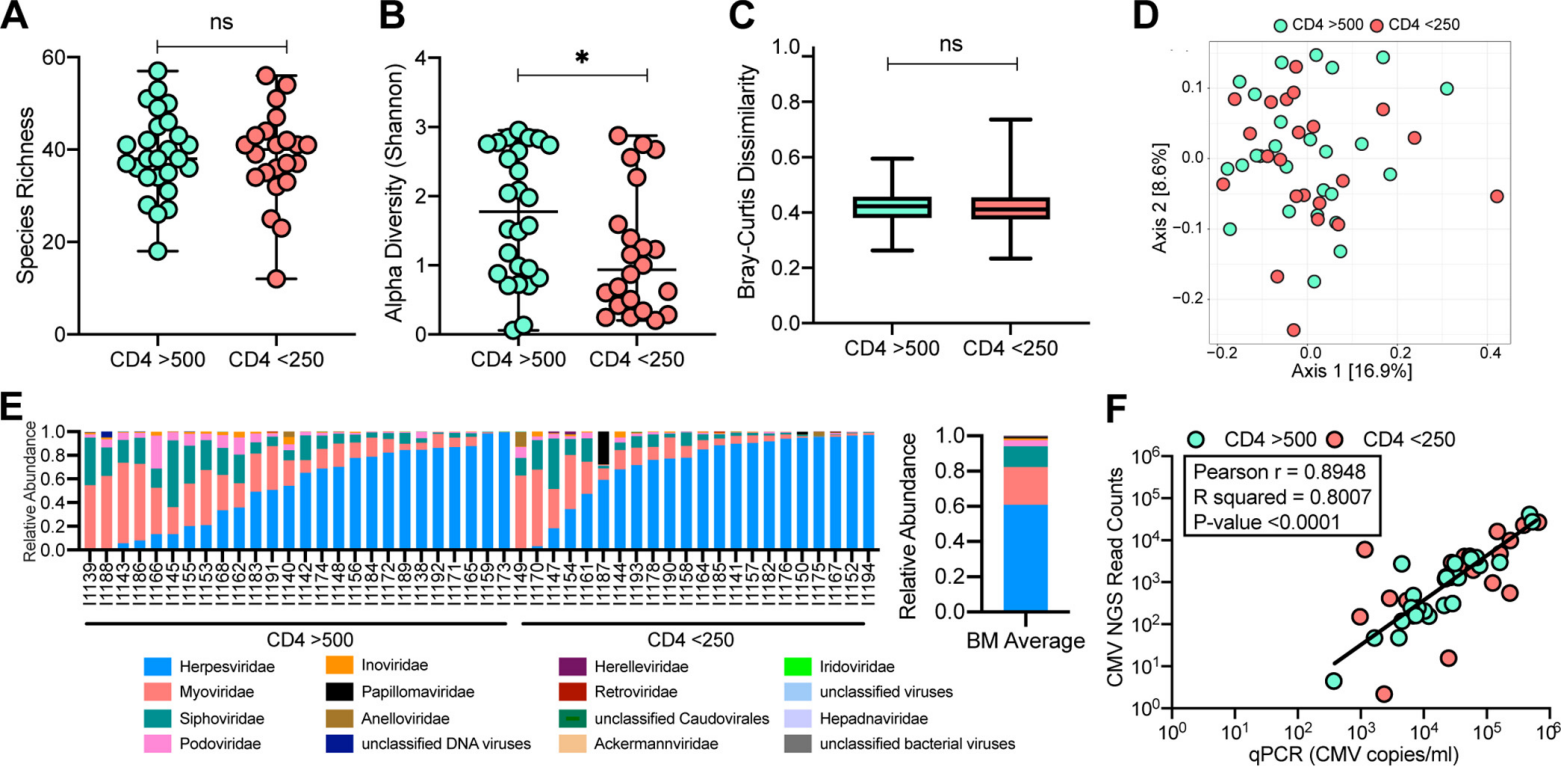
Virome analysis of BM samples from WLHIV by immunosuppression. (A) Richness of viral species in BM samples (median with range). Statistical significance assessed by Mann-Whitney test. (B) Alpha diversity of viral species in BM samples (median with range). Statistical significance assessed by Mann-Whitney test. (C) Unweighted Bray-Curtis distance box plot of BM samples (median with range). Statistical significance assessed by Mann-Whitney test. (D) Unweighted Bray-Curtis PCoA. Colors represent CD4 counts group. (E) Individual and average relative abundance of viruses at family level in BM samples. (F) Plot of CMV sequence reads (log_10_) versus CMV qPCR copy numbers (log_10_). Pearson correlation and linear regression line fit to data.

We next compared the DNA virome in breast milk samples with sufficient sequencing depth (see Materials and Methods) between women with CD4 counts of >500 (*n* = 26) and CD4 of <250 (*n* = 22). The median virome sequence depth between the two groups was not significantly different (544,318 versus 465,067 average reads/sample for CD4 of >500 versus <250, respectively; *P* = 0.32). Breast milk virome richness was similar between the women with high and low CD4 counts (median, 38 versus 40 for CD4 of >500 versus <250, respectively; *P* = 0.95; Fig. 2A). Shannon alpha diversity of the breast milk virome was significantly higher in women with CD4 of >500 than CD4 of <250 (median, 1.77 versus 0.93; *P* = 0.037; Fig. 2B). The beta diversities were not significantly different between the women with high and low CD4 counts (median, 0.42 versus 0.41, respectively; *P* = 0.38; Fig. 2C). PCoA analysis of virome sequence reads showed the two groups were overlapping without any distinct clusters, which was confirmed by PCoA generated by virome contig analysis (Fig. 2D, Fig. S1H). Cytomegalovirus (CMV) dominated the breast milk virome in both groups, and the average relative abundance of CMV sequence reads was higher in women with a CD4 count of <250 versus >500 (70.6% versus 52.9%; *P* = 0.05). CMV sequencing read counts were strongly correlated with CMV genome copies from qPCR measurements (Pearson *r* = 0.89, *P* < 0.0001) (Fig. 2F), validating that CMV abundance by next-generation sequencing (NGS) was reflective of actual viral loads in the breast milk (i.e., not the result of amplification bias). However, CMV viral loads, as measured by qPCR, were not significantly different between CD4 groups (Fig. S1I). Removing the CMV sequences from the virome data set eliminated the difference in alpha diversity (median, 2.77 versus 2.83; *P* = 0.98; Fig. S1J). With the exception of a higher relative abundance of CMV, the other potentially discriminant viruses by CD4 group were very low in abundance (Fig. S1L to N).

### Characteristics of the core breast milk bacterial microbiome and virome in Kenyan women living with HIV.

We found shared features of the bacterial microbiome across the WLHIV in our Kenyan cohort. An average of 49 bacterial families were detected in each sample, the following seven of which had an average relative abundance of >2.5% of sequence reads: *Streptococcaceae* (average relative abundance, 18.8%), *Staphylococcaceae* (10.1%), *Moraxellaceae* (4.1%), *Eubacteriaceae* (3.6%), *Veillonellaceae* (3.0%), *Bacillaceae* (2.8%), and *Enterobacteriaceae* (2.5%; Fig. 1F). While the relative abundance of each bacterial family differed between women, six bacterial families were present in >80% of breast milk samples in our cohort, supporting the presence of a shared core breast milk bacterial microbiome as follows: *Streptococcaceae* (present in 96% of breast milk samples), *Staphylococcaceae* (96%), *Moraxellaceae* (82%), *Eubacteriaceae* (96%), *Bacillaceae* (88%), and *Enterobacteriaceae* (86%).

The breast milk DNA virome was dominated by a high average relative abundance of *Herpesviridae* (60.1%) that was almost exclusively CMV (99.99%), which was found in 98% of breast milk samples in this cohort. Phylogenetic analysis of the assembled CMV genomes demonstrated diverse isolates, arguing against contamination (Fig. S2). Bacteriophages also dominated the virome with a high average relative abundance of *Myoviridae* (21.2%), *Siphoviridae* (11.8%), and *Podoviridae* (3.5%) (Fig. 2E).

10.1128/mSystems.01079-20.2 FIG S2 Phylogenetic analysis of CMV genomes. Phylogenetic tree of concatenated human cytomegalovirus (HCMV) genes (UL 55, UL 144, US 27, US 28) using HCMV5 NCBI reference genomes, CD4 (*n* = 15), and IMM (*n* = 13) breast milk samples. Colors represent the analysis group category of the sample. Download FIG S2, PDF file, 0.3 MB. Copyright © 2021 Maqsood et al. 2021 Maqsood et al. https://creativecommons.org/licenses/by/4.0/ This content is distributed under the terms of the Creative Commons Attribution 4.0 International license.

### Breast milk bacterial microbiome community states and associated bacteriophages.

**FIG 3:**
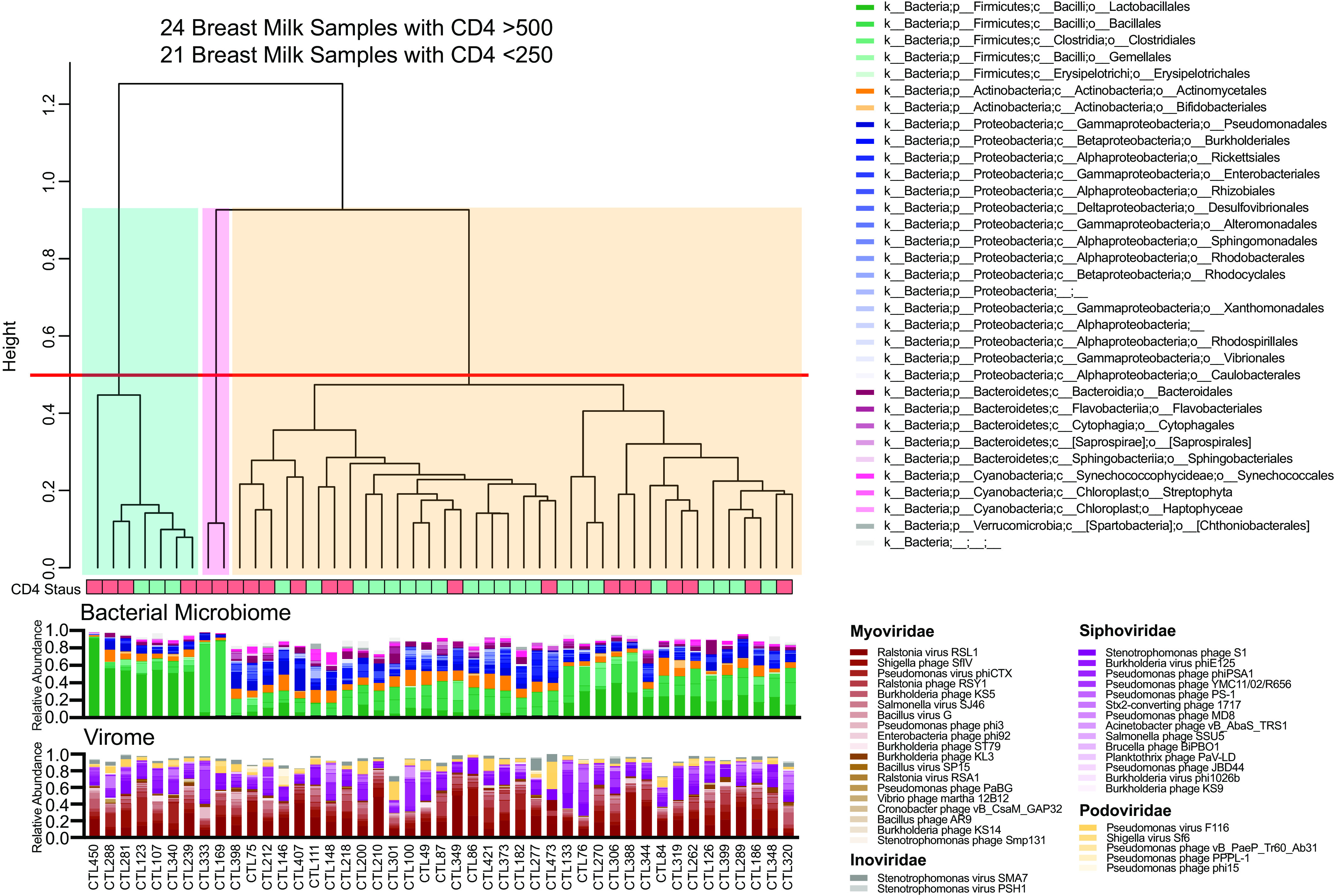
Breast milk microbiome community states. Relative abundance of bacterial at order level, clustered using Euclidean distance. Cutoff of 0.5 applied to separate clusters. Relative abundance of the most frequent phages for the corresponding samples plotted.

Bacterial microbiome communities with similar composition profiles, also called community states, can indicate functional groups as in previous microbiome studies of different sample types (27–29). Breast milk microbiome community profiles were examined by performing Euclidean clustering on the bacterial microbiome data set (at the order taxonomic level). This analysis identified three distinct clusters indicative of bacterial community states (Fig. 3). The largest cluster had similar proportions of *Lactobacillales*, *Bacillales*, *Clostridiales*, *Actinomycetales*, and *Pseudomonadales* across the samples (Fig. 3, yellow cluster), the midsized cluster included seven samples dominated by *Lactobacillales* (blue cluster), and the smallest cluster had only two samples which were both dominated by *Bacillales* (pink cluster).

As bacteriophages were found in similar frequency in the breast milk samples but differed in relative abundance between women, it was hypothesized that individual breast milk DNA virome profiles and bacterial microbiome profiles might influence each other. A number of bacteriophages were found across community states at high abundance, including *Ralstonia* virus RSL1 (average relative abundance, 20.5%), *Shigella* phage SfIV (8.5%), *Pseudomonas* virus phiCTX (5.8%), *Stenotrophomonas* phage S1 (5.8%), *Ralstonia* phage RSY1 (5.7%), and *Burkholderia* virus phiE125 (5.7%), while other phages were present at lower relative abundance of below 5% (Fig. 3, Fig. S3A). However, bacteriophage community state clusters did not correspond to the bacterial community states (Fig. S3B). To validate this, we also assembled and identified bacteriophage contigs using VirSorter. Using these assembled bacteriophage contig sequences, we confirmed that the bacterial microbiome community states did not have distinct bacteriophage profiles (Fig. S3C). We did not find any significant differences in viral loads (plasma HIV, breast milk HIV, or breast milk CMV) between the community states. Taken together, this indicates that breast milk harbors distinct bacterial microbiome community states that are not associated with specific bacteriophage profiles.

10.1128/mSystems.01079-20.3 FIG S3 Breast milk bacterial and viral community state comparison. (A) Euclidean distance dendrogram of bacteriophage data. Cutoff of 0.45 applied to separate clusters. Relative abundance of the most frequent phages for samples plotted. (B) Euclidean distance dendrogram of 16S data at order level matched to samples of dendrogram clustered using Euclidean distance on phage data. (C) Relative abundance of bacterial at order level, clustered using Euclidean distance. Cutoff of 0.5 applied to separate clusters. Relative abundance of the most frequent VirSorter-identified phage contigs for the corresponding samples plotted. Download FIG S3, PDF file, 1.0 MB. Copyright © 2021 Maqsood et al. 2021 Maqsood et al. https://creativecommons.org/licenses/by/4.0/ This content is distributed under the terms of the Creative Commons Attribution 4.0 International license.

### Breast milk virome and infant mortality.

**FIG 4:**
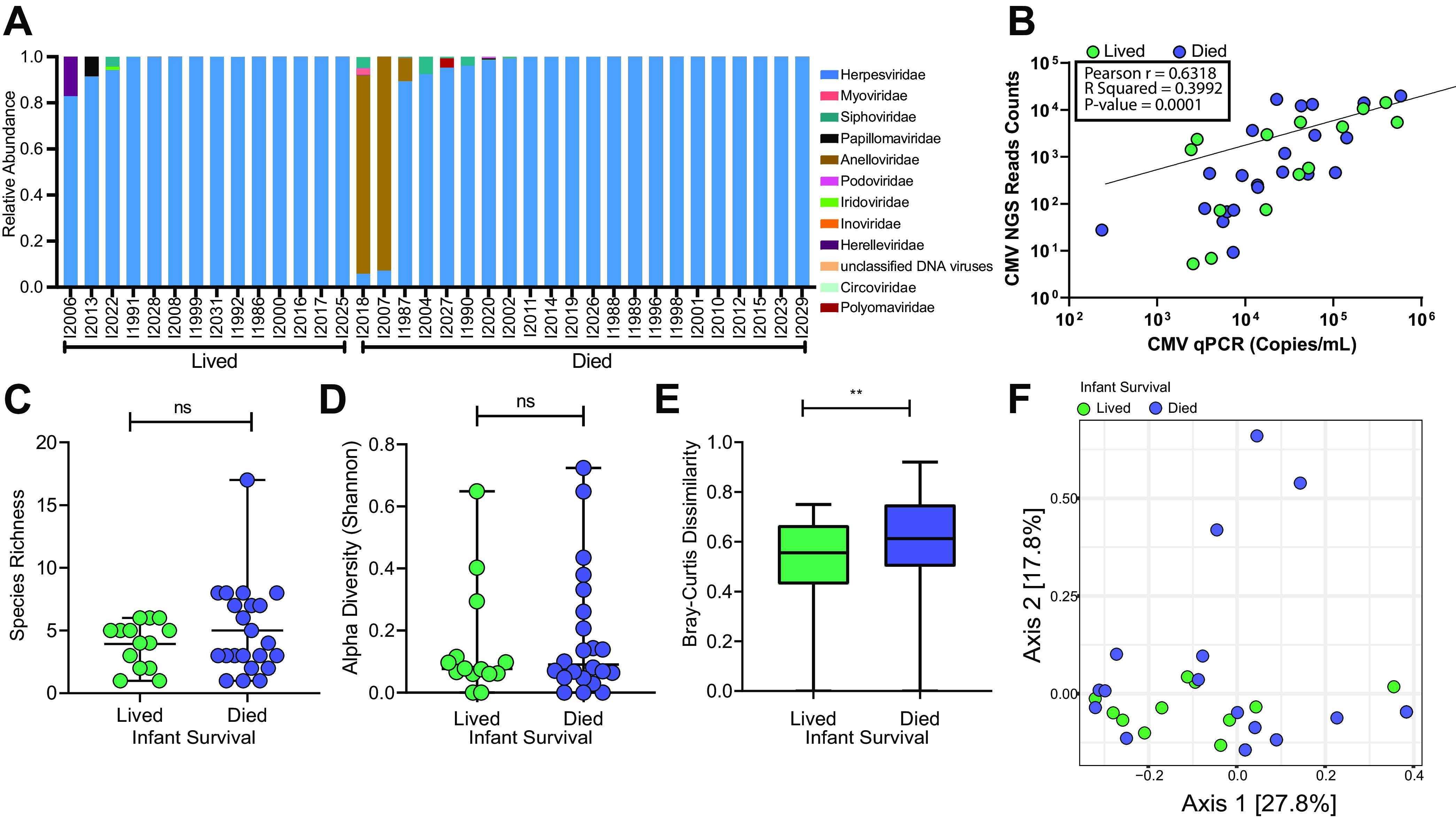
Virome analysis of BM samples from WLHIV by infant mortality. (A) Family-level relative abundance of breast milk virome. (B) Pearson correlation of CMV qPCR copy number and CMV NGS read counts normalized to read depth. (C) Species richness comparing breast milk samples grouped by infant survival (median with range). Statistical significance assessed by Mann-Whitney test. (D) Breast milk virome alpha diversity grouped by infant survival (median with range). Statistical significance assessed by Mann-Whitney test. (E) Box plot comparison of beta diversity (unweighted Bray-Curtis dissimilarity) (median with range). Statistical significance assessed by Mann-Whitney test. (F) PCoA of unweighted Bray-Curtis dissimilarity.

We next sought to evaluate whether the breast milk DNA virome might be associated with infant health outcomes, defined as HIV-infected infants that lived or died during the first 2 years of life. A second set of breast milk samples at 1 month postpartum was sequenced (Table 2). The women in this substudy were transmitting mothers whose infants acquired HIV, and they did not overlap the CD4 substudy above. A total of 45 breast milk samples were sequenced, of which 9 were omitted due to limited sequencing depth, resulting in 14 WLHIV with infants that lived and 22 WLHIV with infants that died. There was insufficient specimen remaining to also conduct 16S bacterial microbiome analyses on these samples. Maternal plasma HIV-1 viral load was significantly higher in the group with infants that died, but not by breast milk HIV-1 viral load (Fig. S4A and B). As in the prior sample set selected for maternal CD4 count (Fig. 2E), the breast milk virome in this sample set selected for infant mortality was also dominated by CMV. However, no significant difference was found in average relative abundance of CMV (97.6% lived and 89.7% died, respectively; *P* = 0.58) or CMV DNA level by quantitative PCR (qPCR) between women whose infants lived versus died (median, 29,218 versus 18,302 copies/ml; *P* = 0.96) (Fig. 4A, Fig. S4C). CMV detected by NGS was correlated with CMV viral load (Fig. 4B). There was no significant difference in the virome species richness (Fig. 4C) or alpha diversity (Fig. 4D) between infant mortality groups. Virome beta diversity was significantly higher in the mothers of infants who died than the mothers of those who lived (0.56 lived versus 0.61 died, *P* = 0.002) (Fig. 4E and F). We found no significant difference in beta diversity when we omit anellovirus sequences (Fig. S4D and E). Although this suggests that the differences may by driven in part by anelloviruses, there was no significant difference in anellovirus levels by qPCR between infant outcome groups (Fig. S4F).

**TABLE 2:** Characteristics of Kenyan WLHIV infant mortality cohort

Infant status^*a*^	Data for infants that:		*P* value
	Lived (*n* = 19)	Died (*n* = 26)	
Maternal age (yr)			
Mean (SD)	24.5 (4.2)	24.6 (4.06)	
Median (IQR)	25 (5)	24.5 (6.3)	0.90
CD4 counts			
Mean (SD)	585.3 (300)	496.8 (185.4)	
Median (IQR)	555 (331)	506 (256.8)	>0.48
CD8 counts			
Mean (SD)	1,168 (729)	1,257 (729)	
Median (IQR)	1,058 (835)	1,167 (672.5)	0.32
BM HIV viral load (copies/ml)			
Mean (SD)	8,742 (15,198)	7,482 (12,780)	
Median (IQR)	880 (6,925)	2,010 (4,844)	0.26
Plasma HIV viral load (copies/ml)			
Mean (SD)	494,702 (1,165,814)	5,046,281 (17,813,193)	
Median (IQR)	65,920 (502,940)	258,570 (660,773)	0.039

a IQR, interquartile range.

10.1128/mSystems.01079-20.4 FIG S4 Supplemental analysis of BM samples from WLHIV with infants that lived or died. (A) Comparison of plasma HIV viral load of WLHIV grouped based on infant survival (median with CI). Statistical significance assessed by Mann-Whitney test. (B) Breast milk HIV viral load grouped by infant survival (median with CI). Statistical significance assessed by Mann-Whitney test. (C) CMV qPCR copy number comparison grouped by infant survival (median with CI). Statistical significance assessed by Mann-Whitney test. (D) Bray-Curtis dissimilarity with Anellovirdae removed (median with Tukey). Statistical significance assessed by Mann-Whitney test. (E) PCoA of Bray-Curtis with Anelloviridae removed. (F) Breast milk anellovirus (ATV) qPCR reads grouped by infant survival (median with CI). Statistical significance assessed by Mann-Whitney test. Download FIG S4, PDF file, 0.2 MB. Copyright © 2021 Maqsood et al. 2021 Maqsood et al. https://creativecommons.org/licenses/by/4.0/ This content is distributed under the terms of the Creative Commons Attribution 4.0 International license.

## DISCUSSION

We investigated the breast milk DNA virome and bacterial microbiome of Kenyan WLHIV that were not on cART and found that breast milk harbors a core bacterial microbiome and a virome dominated by CMV. Notably, this study is unique, as samples were collected during a time when cART was not the standard of care in Kenya, which allowed us to compare women with extreme differences in CD4 count. We found similar bacterial microbiome and DNA virome profiles and diversity across women with high versus low CD4 count and no specific bacteria or viruses that clearly differed based on CD4. Our findings indicate that the breast milk microbiota is highly resilient and is not substantially altered by severe immunosuppression in Kenyan WLHIV.

Previous studies found similar breast milk bacterial microbiomes comparing WLHIV receiving cART and women without HIV infection (14). Taken together with our data from women not on cART, this suggests that the breast milk bacterial microbiome and DNA virome are remarkably resilient to the effects of HIV infection. This resilience is notable, given the effects of immunosuppression that have been observed in the gut and other sites (30, 31). While alterations in the gut microbiome have previously been associated with progressive HIV disease (25, 32), the breast milk bacterial and virome profiles appear to be relatively protected from change. This difference may be due to differing effects of HIV replication at the two sites. In the first weeks of HIV infection, CD4^+^ T cells are dramatically depleted from the gut lamina propria, which is accompanied by profound changes to gut mucosal permeability and resident microbial communities; these changes are only partially reversed by long-term cART. In contrast, breast milk is a continuously replenished and concentrated mixture of innate immune factors originating from the blood or mammary tissues, including antibodies, human milk oligosaccharides, alpha-defensins, cytokines, chemokines, lactoferrin, and others (33). It appears that even in severe immunosuppression, this compartmentalized immune system remains sufficiently intact to preserve the microbiome community structures.

CMV was found in 98% of the samples and dominated the breast milk virome in women with both high and low CD4 counts, with differences in the relative abundance and alpha diversity due to increased CMV in immunosuppressed women. This is consistent with previous studies by our group and others that have reported nearly universal detection of CMV DNA in the breast milk of women with HIV and an inverse association between CD4 counts and breast milk CMV levels (34, 35). Because CMV was recovered from nearly all women in this study, we cannot determine the impact of CMV replication or CMV immune responses on the breast milk bacterial microbiome or virome in our study. However, CMV replication induces host inflammation that could affect reactivation of latent viruses or eubiosis (36), and further research is needed to understand whether it is a causal determinant in the core bacterial microbiome or virome. Whether HIV infection alters the breast milk microbiome and virome will need to be addressed in future studies by comparing WLHIV to uninfected women. Changes in anelloviruses (small circular single-stranded DNA [ssDNA] viruses) detected in the blood and enteric viromes have been implicated with immunosuppression (25, 37, 38). We found that anellovirus was at low abundance in breast milk and was not significantly different between women with CD4 of >500 compared to CD4 of <250 (*P* = 0.35) (Fig. S1K). Similarly, anellovirus levels were not significantly different in infant mortality groups (Fig. S4D). Although virome beta diversity was significantly higher in the mothers of infants who died than those who lived, this was likely driven by three samples with high anellovirus abundance, which when removed, eliminated the differences (Fig. S4E to F).

Given evidence that the breast milk virome contributes to establishment of the infant gut virome (15), we hypothesized that the breast milk virome could affect the infant virome through transmission of pathogenic viruses, transmission of nonpathogenic but potentially inflammatory or immunomodulating viruses, or disruption of protective breast milk immunity for which there is inadequate maternal immune protection from ART-naive women (39). While there was higher beta diversity in the breast milk virome of women whose infants died, we did not find differences in alpha diversity or richness and did not identify any discriminating taxa between the two groups. To reduce confounding, we only included mothers of children that became HIV-infected during follow-up, and thus the results might differ in populations of HIV-exposed but uninfected infants which were not included here. Because viruses and commensal bacteria in breast milk contribute to seeding the infant gut (9), innate immune responses in the infant and/or specific breast milk microbiota may influence the risk of HIV transmission, as has been observed with the vaginal microbiome (40, 41). However, there were only 15 infants in the original cohort that were HIV infected through breast milk transmission, not all of which had maternal breast milk samples available, and thus we were not able to examine the association between breast milk virome and HIV transmission. Because these specimens were not prospectively collected for microbiome studies and had been used for other virology and immunology studies, our sample size was limited, and we did not have adequate specimen volumes remaining to examine the association between breast milk bacterial microbiome and infant mortality. In addition, our study design was cross-sectional and limited to analysis of maternal samples at 1 month postpartum. Longitudinal studies are needed which adjust for confounding variables such as preterm birth, age of infant death, and malnutrition and that include infant samples to address whether maternal HIV affects maternal-infant transmission.

In summary, this study advances our fundamental understanding of the breast milk core microbiome and virome in the context of HIV disease in Kenya and demonstrates remarkable resilience of the microbiome to the effects of severe HIV-induced immunosuppression. More research is needed to identify the determinants of breast milk virome diversity and composition and the potential impact of CMV on microbiome and virome diversity.

## MATERIALS AND METHODS

### Study population.

Breast milk samples were collected from women enrolled in the CTLs and Prevention of HIV-1 Transmission Study, in Nairobi, Kenya, between 1999 and 2002 (42). As described previously, women enrolled during pregnancy and were followed together with their infants for 2 years postpartum. As was the standard of care at the time of the study, mothers were given short-course zidovudine during their last trimester to reduce the risk of mother-to-child transmission but received no other cART during follow-up. In addition, exclusive breastfeeding was recommended for most WLHIV in sub-Saharan Africa because replacement feeding was not feasible, affordable, or safe for most women (43–45), and all women in this analysis breastfed their infants. The substudy presented here describes two cross-sectional sample sets and analyses of women from this cohort. In the first substudy, we selected all participants who had a breast milk sample available from 1 month postpartum and had CD4 counts of >500 cells/mm^3^ (*n* = 30) or <250 cells/mm^3^ (*n* = 23). In the second substudy, we selected available breast milk samples, 1 month postpartum, from mothers of HIV-infected infants that died (*n* = 26) or survived (*n* = 19) until age 2 years. These sample sizes resulted in 80% power to detect a 0.78 and 0.85 difference in Shannon diversity index in the CD4 and infant mortality substudies, respectively. This study was approved by the University of Nairobi/Kenyatta National Hospital Ethics and Research Committee, the University of Washington, The Fred Hutchinson Cancer Research Center, and Arizona State University Institutional Review Boards; all women provided written informed consent for study participation and for storage and use of their specimens in future studies of HIV.

### Virome and bacterial microbiome 16S rRNA sequencing.

Breast milk samples were vortexed briefly, centrifuged at 13,500 rpm for 10 min, and separated into pellet (bacterial microbiome analysis) and supernatant (virome analysis). From the pellet, DNA extraction was performed using a DNeasy PowerSoil kit (Qiagen). From the supernatant fraction, total nucleic acid extraction was performed using the eMAG instrument (bioMérieux) according to the manufacturer’s guidelines. Negative controls consisting of phosphate-buffered saline (PBS) spiked with *Enterobacteria* phage λ were extracted in parallel to the breast milk samples to assess contamination during extraction. Additionally, controls of PBS spiked with lambdavirus DNA were used to assess cross-sample contamination during amplification and sequencing. Bacterial and virome sequencing were performed as previously described (46, 47). Briefly, PCR was performed on the DNA from the DNeasy eluate using primers for the V4 region (F515/R806) for bacterial microbiome sequencing, and total nucleic acid from the eMAG eluate was amplified with GenomiPhi V2 (GE Healthcare) for virome sequencing. Libraries were sequenced on the Illumina MiSeq platform (version 2, 2 × 250) at the ASU Biodesign Institute Genomics Core facility.

### Bacterial microbiome 16S rRNA analysis.

Illumina sequencing reads (2 × 250 paired-end reads; average, 29,818 ± 24,604 reads per sample) were processed through QIIME 2 using DADA2 to obtain denoised sequence reads and amplicon sequence variants (ASV) (48). To remove contaminant ASVs, the Decontam (version 1.2.1) package in R, which uses the “prevalence” method to identify contaminants, was applied at threshold 0.25 (Fig. S5A) (49). Additionally, two ASVs found in high abundance and similar proportions in participant samples and PBS were removed. To control for intersample depth variability, QIIME 2 was used to rarefy data to 8,000 reads (subsampling without replacement) and create a phylogenetic tree and taxonomy using the GreenGenes database (Fig. S5B). Out of 53 women’s samples sequenced, 3 samples were omitted due to limited sequencing depth; 28 samples with CD4 counts of >500 and 22 samples with CD4 of <250 were analyzed. We performed core ecological metrics of alpha diversity, unweighted UniFrac distances, and weighted UniFrac distances using QIIME 2. PCoA was plotted using ggplot2 (version 3.3.1) in R (https://ggplot2.tidyverse.org; 50).

10.1128/mSystems.01079-20.5 FIG S5 Analysis of NGS dataset assessing for contamination sequences. (A) Relative abundance of 16S contaminants for breast milk samples and PBS buffer negative controls in the CD4 substudy, shown by Decontam threshold (top) and lineage (bottom). Buffer negative controls (red), samples sequenced by omitted after QC (purple), and samples analyzed (green) are shown. (B) Rarefaction plot of average bacterial ASV richness and alpha diversity for CD4 of >500 and CD4 of <250 samples at increasing depth. (C) Unweighted Bray-Curtis PCoA of breast milk virome samples and PBS buffer with unnormalized and unfiltered data. (D) Relative abundance of virome contaminants for breast milk samples and PBS buffer negative controls in the CD4 substudy, shown by Decontam threshold (top) and lineage (bottom). Buffer negative controls (red), samples sequenced by omitted after QC (purple), and samples analyzed (green) are shown. (E) Relative abundance of virome contaminants for breast milk samples and PBS buffer negative controls in the infant mortality substudy, shown by Decontam threshold (top) and lineage (bottom). Buffer negative controls (red), samples sequenced by omitted after QC (purple), and samples analyzed (green) are shown. (F) Proportion of viral contigs that are identified in viral RefSeq + Neighboring sequences database using BLASTx (blue) and those that were not (gray). Download FIG S5, PDF file, 0.6 MB. Copyright © 2021 Maqsood et al. 2021 Maqsood et al. https://creativecommons.org/licenses/by/4.0/ This content is distributed under the terms of the Creative Commons Attribution 4.0 International license.

### Virome analysis.

Illumina sequencing reads (2 × 250 paired-end reads; average, 1.40 ± 0.75 million reads per sample) were quality filtered to remove adapters and low-quality bases using BBTools (version 37.64) (51). High-quality-filtered reads were queried against the viral RefSeq + Neighboring sequences database (downloaded March 2019) using BLASTx to identify viral reads. The taxonomic lineage of viral reads was assigned using the MEGAN (version 6.18.0) naive lowest common ancestor (LCA) algorithm with the parameters min support = 1, min support percentage = 0, and bitscore of 100 (52). Contamination was assessed by an unweighted Bray-Curtis PCoA plot clustering the breast milk samples separately from the PBS samples and Decontam at a threshold of 0.25 (Fig. S5C to E). Samples were removed if they had sequence reads below 168,000 (CD4 analysis) or 50,000 (infant mortality analysis), to which the data were then normalized, or if they had viral reads of <250. Virus species with less than 1 normalized read were masked to remove low-abundance species. In the CD4 substudy, out of 53 women’s samples sequenced, 5 samples were omitted due to quality control (QC) described above, 26 samples with CD4 counts of >500 and 22 samples with CD4 of <250 were analyzed. In the second infant mortality substudy, out of 45 women sequenced, 9 samples were omitted due to QC described above, 22 samples from mothers of HIV-infected infants that died and 14 that survived were analyzed. Ecological analyses (richness, alpha diversity-Shannon Index) were performed using vegan R package (version 2.5-5) (53). The unweighted Bray-Curtis beta diversity distances and PCoA plots were obtained by applying QIIME 2 and R package ggplot2 on a presence-absence species data set.

For validating the reads-based analysis, we built contigs using IDBA-UD with quality-filtered reads for each sample and used minimus 2 to merge any overlapping contigs (54, 55). The contigs of each sample were concatenated and were filtered to remove duplicates (minidentity = 99) and contigs with a length of <800 kb using bbtools. These contigs were then used to identify bacteriophages using VirSorter (parameters –virome, -db 1) (version 1.0.6), and the identified contigs were then used to build a database (54, 56). Quality-filtered reads for each sample were mapped to the VirSorter contig database using the Burrows-Wheeler Aligner MEM algorithm (BWA-MEM; -M -L 95,95) (version 0.7.17-r1188) to validate the cohort trends with PCoA plots (38). To determine the proportion of viral contigs (VirSorter) that were not identifiable (i.e., viral “dark matter”), we queried the viral contigs against the viral RefSeq + Neighboring sequences database using BLASTx (E value, 1E^−3^) (Fig. S5F).

### qPCR assays.

CMV qPCR was performed in a QuantStudio 3 (Applied Biosystems) PCR system in 20-μl reaction mixtures containing 10 μl of TaqMan fast universal PCR 2X master mix (Applied Biosystems), 1.8 μl (10 μM) of each primer (HCMV-FOR, 5′-TGGGCGAGGACAACGAA-3′, and HCMV-REV, 5′-TGAGGCTGGGAAGCTGACAT-3′), 0.5 μl (10 μm) probe (5′ 56-FAM/TGG GCA ACC/ZEN/ACC GCA CTG AGG/3IABkFQ/-3′), 0.9 μl water, and 5 μl of extracted total nucleic acid (TNA) (57). The assay was performed with preliminary denaturation for 20 s at 95°C (slope, 4.14°C/s), followed by 40 cycles of denaturation at 95°C for 1 s (slope, 4.14°C/s) and annealing at 60°C for 20 s (slope, 3.17°C/s). A standard curve was generated using synthesized double-stranded DNA gene fragment (IDT gBlocks) of the CMV genome region from genome position 82468 to 82948 in reference to CMV complete genome GenBank accession number MT044484.1. Analysis was performed with a 0.04 threshold determined by the standard curve; detection measured by the VIC reporter and NFQ-MGB Quencher.

Anellovirus qPCR was performed in 25-μl reaction mixtures containing 12.5 μl of TaqMan fast universal PCR master mix (2XApplied Biosystems), 2.3 μl (10 μM) of each primer (ATV-FOR, 5′-GTGCCGIAGGTGAGTTTA-3′, and ATV-REV, 5′-AGCCCGGCCAGTCC-3′), 0.7 μl (10 μm) probe (5′ 56-FAM/TCAAGGGGCAATTCGGGCT/36-TAMSp/-3′), 4.2 μl water, and 3 μl of extracted TNA. The assay was performed with preliminary denaturation for 20s at 95°C (slope, 4.14°C/s), followed by 40 cycles of denaturation at 95°C for 1 s (slope, 4.14°C/s) and annealing at 60°C for 20 s (slope, 3.17°C/s). A standard curve was generated using a double-stranded gene fragment cloned an anellovirus genome corresponding to the genome sequence of position 1694 to 1756 in reference to Anellovirus complete genome GenBank accession number MH649099.1. Analysis was performed with a 0.03 threshold as determined by the standard curve, measuring FAM reporter and NFQ-MGB Quencher.

### Statistical analysis.

Since sample selection criteria were based on women with CD4 counts of >500 or <250 and women with HIV-infected infants that lived or died within 2 years of life, we treated the data as ordinal. Additionally, since the data follow a nonnormal distribution, statistical significance of virome diversity and bacterial 16s diversity measures comparing groups of women based on CD4 or infant mortality was assessed using a nonparametric Mann-Whitney U test (two-tailed). Pearson correlation was performed between CMV sequence read counts from the virome sequencing and the CMV qPCR copy numbers. The Mann-Whitney U test (two-tailed) was used to determine significance between qPCR levels in groups of women.

### Cooccurring bacterium and bacteriophage analysis.

The Euclidean distance between the rarefied bacterial microbiome sequences at the order level was computed and used to cluster the samples. The relative abundance of each order of bacteria was plotted using the clustering order, and a cutoff of 0.5 was used to determine the breast milk community states. To evaluate trends of the phages in the 16S clusters, the relative abundances of the top phage species (as chosen by average and frequency across breast milk samples) were plotted for each sample to assess virome profiles within community states. Additionally, the community states of viromes were also clustered separately using Euclidean distance on the phage data set. We also corroborated the phage trend by plotting the top VirSorter identified bacteriophage contigs according to community states. We performed a one-way nonparametric analysis of variance (ANOVA) test (Kruskal-Wallis and Dunn’s multiple-comparison test) to assess whether community states were associated with viral loads (copies/ml) of plasma HIV, breast milk HIV, or breast milk CMV. For cooccurrence analyses, 45 WLHIV had data for both virome and bacterial microbiome, 24 samples with CD4 counts of >500 and 21 samples with CD4 of <250.

### Phylogenetic analysis.

Contigs were assembled from quality-filtered sequencing reads with metaSPAdes (version 3.14.0) for each individual breast milk sample specimen (58). CMV contigs were extracted by mapping against the human herpesvirus 5 strain Merlin (GenBank Accession number NC_006273) using BWA. The CMV UL55, UL144, US27, and US28 genes were annotated and concatenated in Geneious Prime (version 2020.0.5) (59). Multiple sequence alignment was performed with CMV sequences and 11 reference genomes (NCBI GenBank accession numbers FJ616285, GQ221975, KJ872542, KT959235, KJ361946, KJ361959, JX512198, KU550090, KR534213, HQ380895, and NC_006273) using MAFFT (default parameters) (60). The sequence alignment was trimmed using trimAl (default parameters) (61). Statistical selection of best-fit models was determined using jModelTest 2 (g 4 -i -f -BIC -a) (62). Maximum likelihood (ML) phylogenetic trees were constructed using PhyML (K80 +I +G, ti/tv 3.8561) (63). Support for ML trees was assessed by 1,000 nonparametric bootstraps.

### Data availability.

Sequence data have been deposited in the NCBI under the BioProject number PRJNA635738. The code script to reproduce the analysis and plots is available in the supplemental material (CD4_Data_Script).

10.1128/mSystems.01079-20.6 Text S1 Code scripts to reproduce the analysis and plots. Download Text S1, DOCX file, 0.01 MB. Copyright © 2021 Maqsood et al. 2021 Maqsood et al. https://creativecommons.org/licenses/by/4.0/ This content is distributed under the terms of the Creative Commons Attribution 4.0 International license.
