# Supplementary figures and images for: Breast Milk Virome and Bacterial Microbiome Resilience in Kenyan Women Living with HIV
Source: mSystems. 2021 Mar 16;6(2):e01079-20. doi: 10.1128/mSystems.01079-20 (PMC8546991; doi:10.1128/mSystems.01079-20)

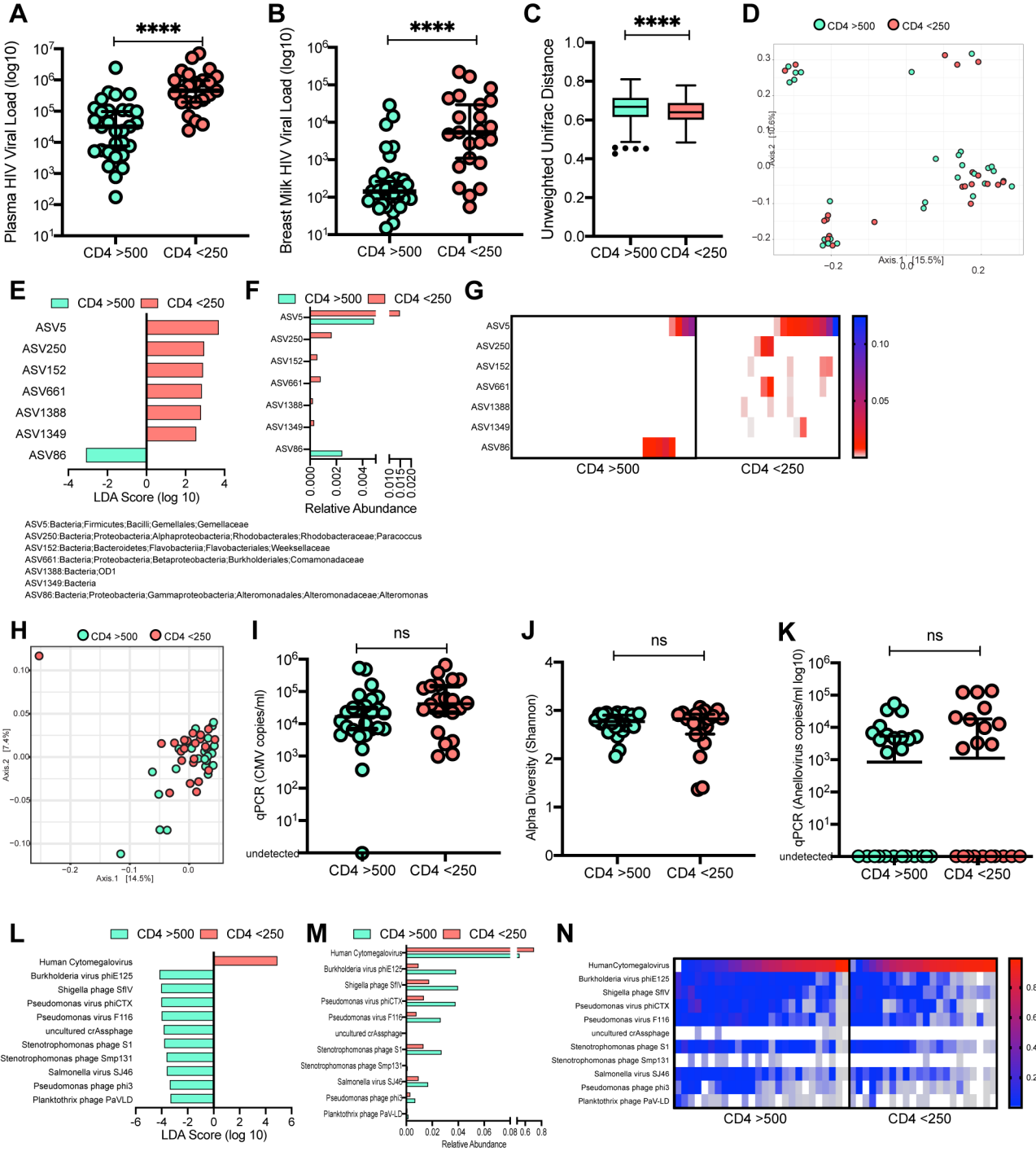

Supplement: FIG S1 [file msystems.01079-20-sf001.pdf]

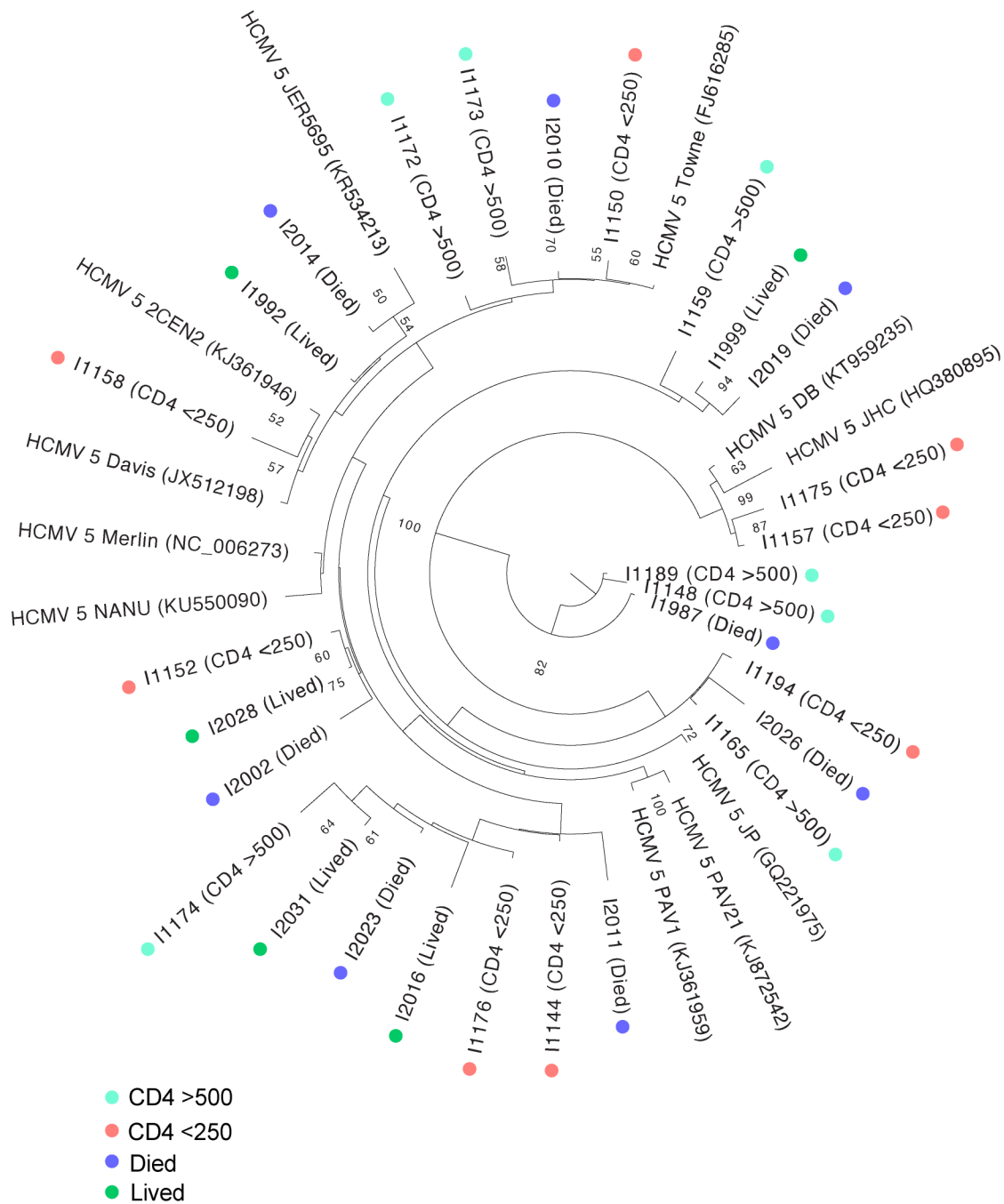

0.02

Supplement: FIG S2 [file msystems.01079-20-sf002.pdf]

**A**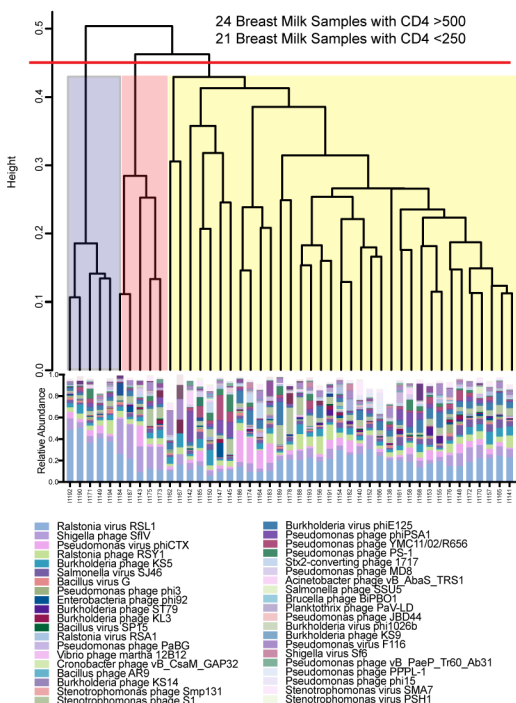**C**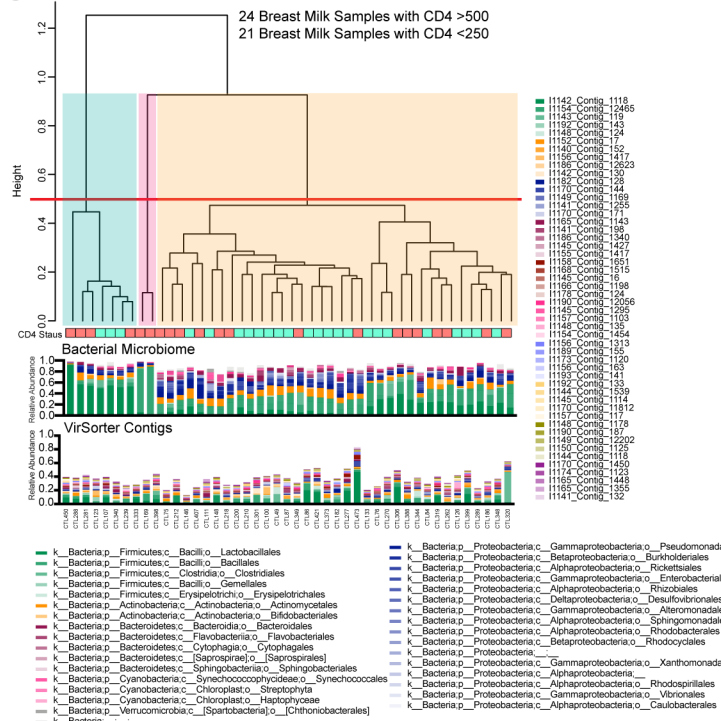**B**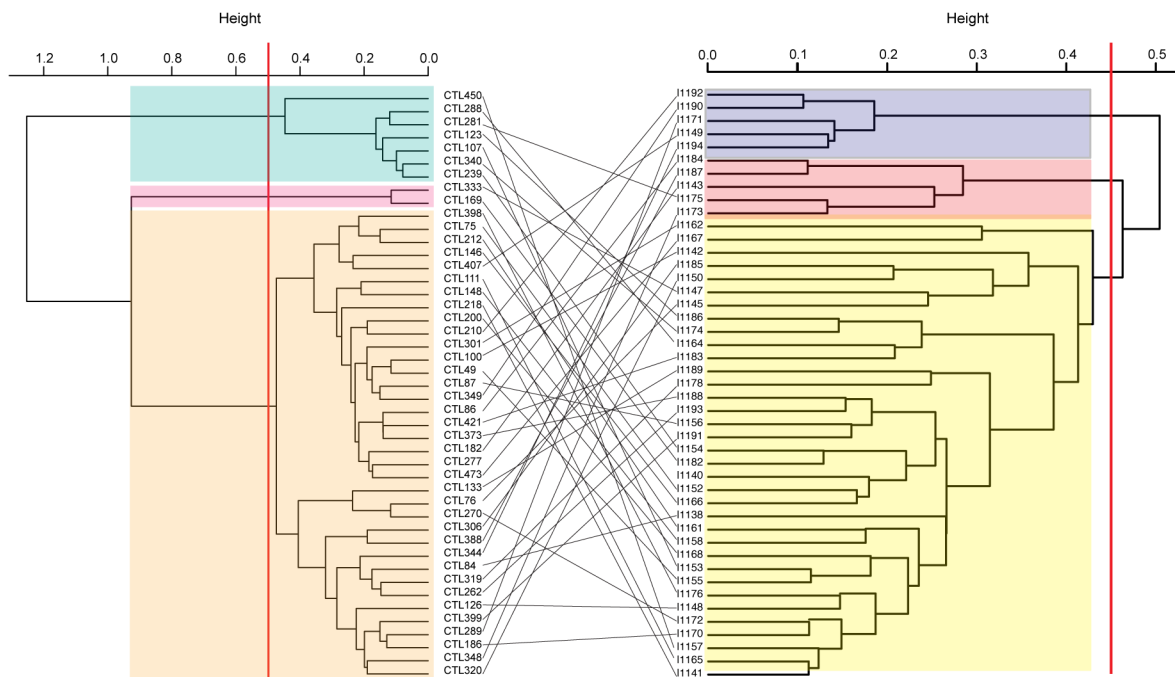

Supplement: FIG S3 [file msystems.01079-20-sf003.pdf]

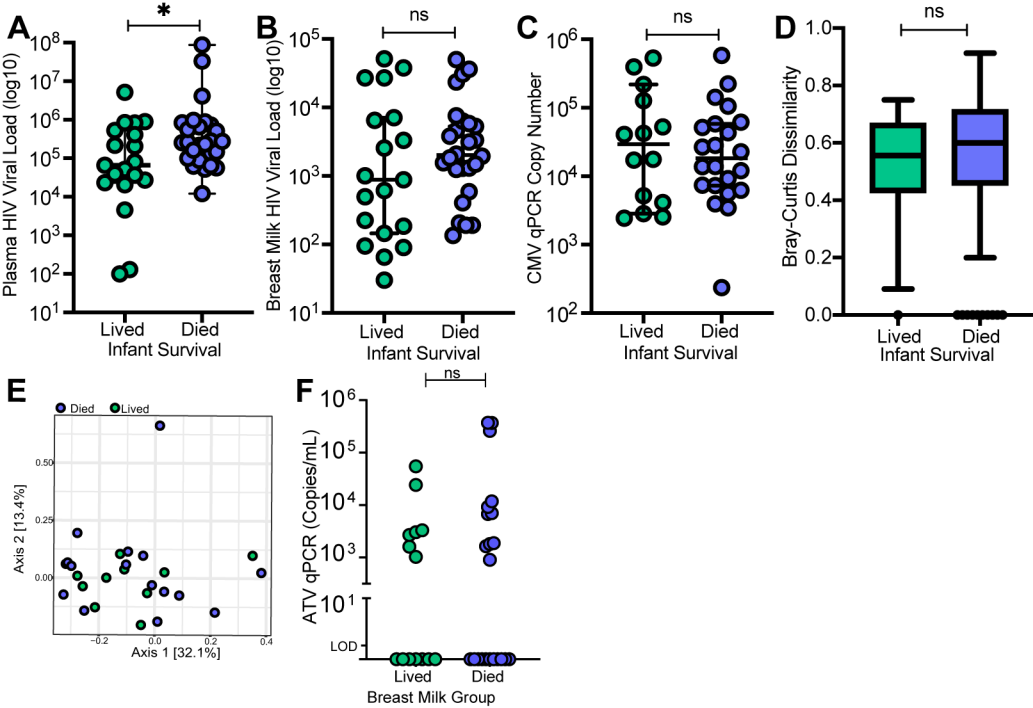

Supplement: FIG S4 [file msystems.01079-20-sf004.pdf]

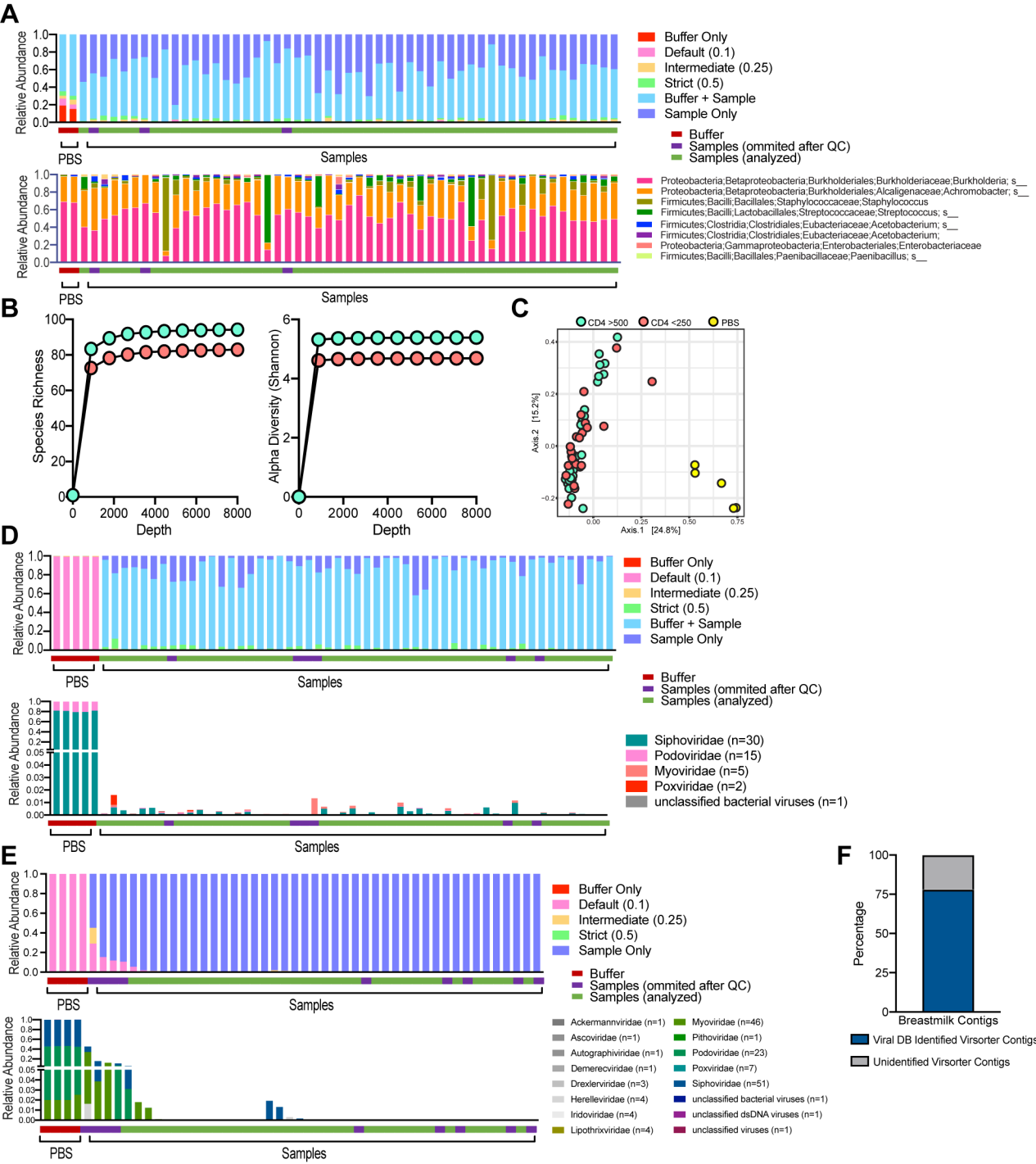

Supplement: FIG S5 [file msystems.01079-20-sf005.pdf]
