# Supplementary material for: Breast Milk Virome and Bacterial Microbiome Resilience in Kenyan Women Living with HIV
Source: mSystems. 2021 Mar 16;6(2):e01079-20. doi: 10.1128/mSystems.01079-20 (PMC8546991; doi:10.1128/mSystems.01079-20)
Supplement: Text S1 [file msystems.01079-20-s0001.docx]

**Code script**

library(phyloseq)

library(randomForest)

library(Boruta)

library(varSelRF)

library(decontam)

library(ggplot2)

library(vegan)

library(ape)

library(taxonomizr)

library(gplots)

library(pROC)

library(biomformat)

library(tidyverse)

library(readxl)

#############PreDecontamVirome#############

rawData <- read_excel("Documents/CTL/Figures In Progress/Figures_Rearrage_v2/ForPaper/CD4ViromeData.xlsx",sheet = "RawData", col_types = c("skip","text",

"numeric", "numeric", "numeric", "numeric", "numeric", "numeric",

"numeric", "numeric", "numeric", "numeric", "numeric", "numeric", "numeric", "numeric", "numeric",

"numeric", "numeric", "numeric", "numeric", "numeric", "numeric","numeric", "numeric", "numeric",

"numeric", "numeric", "numeric", "numeric", "numeric", "numeric","numeric", "numeric", "numeric",

"numeric", "numeric", "numeric", "numeric", "numeric", "numeric", "numeric", "numeric", "numeric",

"numeric", "numeric", "numeric", "numeric", "numeric", "numeric", "numeric", "numeric", "numeric",

"numeric", "numeric", "numeric", "numeric", "numeric", "numeric", "numeric"))

metadata <- read_excel("Documents/CTL/Figures In Progress/Figures_Rearrage_v2/ForPaper/CD4ViromeData.xlsx", sheet = "Metadata")

rawData2<-rawData %>% remove_rownames %>% column_to_rownames(var="speciesNAmes")

set.seed(100)

data<-as.matrix(t(rawData2))

contam<-isContaminant(data, method = 'prevalence', neg =metadata$isNeg, threshold=0.1)

contam.intermediate<-isContaminant(data, method = 'prevalence', neg =metadata$isNeg, threshold=0.25)

contam.strict<-isContaminant(data, method = 'prevalence', neg =metadata$isNeg, threshold=0.5)

write.csv(contam, "CTL_SpeciesDefault.csv")

write.csv(contam.intermediate,"CTL_SpeciesIntermediate.csv")

write.csv(contam.strict,"CTL_SpeciesStrict.csv")

rawData3<-rawData2

rawData3[rawData3 > 0] <- 1

feature_table <- as.data.frame(rawData3)

dim(feature_table)

OTU=otu_table(feature_table,taxa_are_rows =TRUE)

sample_names(OTU)

metadata<-import_qiime_sample_data(metadata)

sample_names(metadata)

physeq = phyloseq(OTU,metadata)

ord <- ordinate(physeq,

method = "PCoA",

distance = "bray") ##Bray Curtis

#5. Plot by Disease Group

BrayCurtis_DiseaseGroup <- plot_ordination(physeq = physeq,

ordination = ord,

shape = "CD4Cat", # phyloseq metadata variable

color = "CD4Cat", # phyloseq metadata variable

axes = c(1,2),

title='Bray Curtis Axis1 vs Axis2') +

theme_bw() +

theme(text=element_text(size=20))+

geom_point(size = 8)+ #,alpha=0.5) +

scale_color_manual(values=c("#66FFCC","#FF6666","Yellow"))+

scale_shape_manual(values = c( 19, 19,19))

BrayCurtis_DiseaseGroup

#############PostDecontamVirome#############

cleanData <- read_excel("Documents/CTL/Figures In Progress/Figures_Rearrage_v2/ForPaper/CD4ViromeData.xlsx",sheet = "168KNormalized_cleanSpeciesData")

cleanData2<-cleanData %>% remove_rownames %>% column_to_rownames(var="speciesNAmes")

cleanData3<-cleanData2

cleanData3[cleanData3 > 0] <- 1

metadata <- read_excel("Documents/CTL/Figures In Progress/Figures_Rearrage_v2/ForPaper/CD4ViromeData.xlsx", sheet = "Metadata")

library(vegan)

dataTransposed<-t(cleanData2)

data_vegan<-diversity(dataTransposed, index = 'shannon')

write.table(data_vegan,"2019_SpecieCTL_AlphaDiversity.txt", sep = '\t')

dataTransposed<-t(cleanData3)

dis <- vegdist(dataTransposed, method = "bray")

dis2<-as.matrix(dis)

write.table(dis2,"2019_SpecieCTL_BrayCurtis.txt", sep = '\t')

feature_table <- as.data.frame(cleanData3)

dim(feature_table)

OTU=otu_table(feature_table,taxa_are_rows =TRUE)

sample_names(OTU)

metadata<-metadata

sample_names(metadata2)

physeq = phyloseq(OTU,metadata)

ord <- ordinate(physeq,

method = "PCoA",

distance = "bray") ##Bray Curtis

#5. Plot by Disease Group

BrayCurtis_DiseaseGroup <- plot_ordination(physeq = physeq,

ordination = ord,

shape = "CD4Cat", # phyloseq metadata variable

color = "CD4Cat", # phyloseq metadata variable

axes = c(1,2),

title='Bray Curtis Axis1 vs Axis2') +

theme_bw() +

theme(text=element_text(size=20))+

geom_point(size = 8)+ #,alpha=0.5) +

scale_color_manual(values=c("#66FFCC","#FF6666"))+

scale_shape_manual(values = c( 19, 19))

BrayCurtis_DiseaseGroup

#############BacterialCommunityState#############

rawData<- read_excel("Documents/CTL/Figures In Progress/Figures_Rearrage_v2/ForPaper/CD4BacterialMicrobiomeData.xlsx", sheet = "rawdata")

metadata <- read_excel("Documents/CTL/Figures In Progress/Figures_Rearrage_v2/ForPaper/CD4BacterialMicrobiomeData.xlsx", sheet = "Metadata")

rawData2<-rawData %>% remove_rownames %>% column_to_rownames(var="#OTU ID")

set.seed(100)

data<-as.matrix(t(rawData2))

contam<-isContaminant(data, method = 'prevalence', neg =metadata$isNeg, threshold=0.1)

contam.intermediate<-isContaminant(data, method = 'prevalence', neg =metadata$isNeg, threshold=0.25)

contam.strict<-isContaminant(data, method = 'prevalence', neg =metadata$isNeg, threshold=0.5)

write.csv(contam, "CTL_SpeciesDefault.csv")

write.csv(contam.intermediate,"CTL_SpeciesIntermediate.csv")

write.csv(contam.strict,"CTL_SpeciesStrict.csv")

library(dendextend)

CD4BacterialMicrobiomeData <- read_excel("Documents/CTL/Figures In Progress/Figures_Rearrage_v2/ForPaper/CD4BacterialMicrobiomeData.xlsx", sheet = "communityState.1")

CommunityStateData<-CD4BacterialMicrobiomeData %>% remove_rownames %>% column_to_rownames(var="External ID")

CommunityStateData.1<-t(CommunityStateData)

hca <- hclust(dist(CommunityStateData.1,method = 'euclidean'))

plot(hca, hang = -1, cex = 0.6)
